# Sources and content of popular online videos about autism spectrum disorders

**DOI:** 10.15171/hpp.2017.41

**Published:** 2017-09-26

**Authors:** Betty Kollia, Margaret T. Kamowski-Shakibai, Corey H. Basch, Ashley Clark

**Affiliations:** ^1^Department of Communication Disorders and Sciences, William Paterson University of New Jersey, Wayne, NJ 07470, USA; ^2^Department of Communication Sciences and Disorders, Marymount Manhattan College, New York, 10021, USA; ^3^Department of Public Health, William Paterson University of New Jersey, Wayne, NJ 07470, USA

**Keywords:** Autism spectrum disorder, Developmental delay, Communication disorders, Videos, Social media, YouTube

## Abstract

**Background:** Our study aimed to determine source of upload and content portrayed in the100 most-viewed videos on autism spectrum disorders (ASDs) on the video sharing public forum, YouTube. ASDs have become highly prevalent in the last decade, arousing a significant response from the media and psycho-educational health professions. Utilization of and reliance on social media for information on health matters has also proliferated. Some suggest that online videos could promote early detection (and intervention) of ASD by prompting caregivers to seek guidance. However, the usefulness of the available videos is unclear.

**Methods:** The 100 most popular YouTube videos were examined for source of upload and information provided. Popularity was determined by number of views, using the filter tool.

**Results:** The videos had more than 121 million views combined. Only one video had been uploaded by a professional (a clinical psychologist). The 99 (non-professional) videos provided minimal data and research into known ASD risk factors. Interestingly, discredited vaccine-associated risks were promoted in 16% (95% CI = 09%–25%) of the 100 videos analyzed. Many videos featured a child with ASD exhibiting some characteristic patterns, such as engaging in a repetitive behavior (73%, 95% CI = 63%-81%); about as many videos referenced various therapies (75%, 95% CI = 65%-83%); and 54% (95% CI = 44%–64%) and 61% (95% CI =51%–71%) of the videos mentioned the economic and emotional toll of ASD on the family,respectively. Additional variables are discussed.

**Conclusion:** The most popular online videos were primarily uploaded by non-professionals and provided limited content regarding ASD. Given the wide reach of social media and its potential for providing valuable information and guidance to the public on matters pertaining to ASD, we wish to underscore the necessity for a professional presence in this medium.

## Introduction

The prevalence of autism spectrum disorders (ASDs) has more than doubled in the last 15 years. The United Nations (UN) has given an “official day” designation to only four health conditions, one of which is World Autism Awareness Day. In 2016, a report issued by the Autism and Developmental Disabilities Monitoring (ADDM) Network of the Centers for Disease Control and Prevention (CDC) reported that on average, in 2012, about 1 in 68 children was diagnosed with ASD (14.6 per 1000).^1^ In the surveillance report for a decade prior to that (2002), the average prevalence was 1 in 150 children (or 6.6 per 1000).^2^

With the rise in rates of diagnoses of ASD, and the availability, popularity, and focus of mainstream and social media, the awareness of ASD and its many faces have emerged. The search engine result of the keyword “autism” is 89 million. Given the plethora of available sources of information as well as the overload and stress that these “searches” can create,^3^ many look to more “compact” information, such as that which is conveyed via video. A search for “autism” via YouTube results in 1 780 000 videos, representing 0.02% of the search engine’s results. Moreover, feature length movies starring popular actors have helped in raising the public’s awareness of autism as well as made-for-TV movies, TV shows and series, and special reports in news programs.

Film and video may be the preferred medium for learning about and understanding topics of interest. Much like other parents who have a child diagnosed with ASD, film critic Leslie Felperin^4^described her experiences aimed at learning about her child’s diagnosis through both written material and videos. She expounded on some of the shortcomings of depictions in some movies, (which insisted on portraying persons with ASD as having a sliver of genius that acts as a catalyst in the movie, i.e., Rain Man^5^, Mercury Rising,^6^ The Accountant,^7^ and others), and the improvements in others, such as the biographical film on Temple Grandin.^8^

Increasingly, not only parents, but also those who may be interacting with a young child whose behavior may be unlike what is expected conduct searches for information on ASD. These include early childhood educators, teachers, family friends, even pediatricians. As the public is looking for easily digestible information on ASD, researchers have sought to assess people’s familiarity with ASD. Harrison et al^9^ examined 67 peer-reviewed articles worldwide that surveyed ASD knowledge of primarily health and education professionals. Only 7% of the studies analyzed used a metric with psychometric power. The authors of this meta-analytic study found the cross-study information difficult to synthesize. These findings pointed to the necessity of designing and using a unified, “golden” measure of ASD knowledge that has psychometric validity and cultural appropriateness. Understanding awareness and knowledge of ASD is important to tailor information provided to the public. Health professionals must consider the gaps in knowledge and misconceptions of ASD epidemiology, diagnostic criteria, symptomatology, therapeutic interventions, and educational services available. Most importantly, health professionals must carefully consider the platform used to convey this information to ensure that the public receives the necessary information. Although the CDC has information tailored to the specific questions of families, educators, healthcare providers, and partners,^10^ freely accessible videos on the internet can be simple, informative, and useful, and hence may be the most frequent choice of consumers.

Researchers have identified potential contributors to the appearance of ASD as well as medical comorbidities.^11^ Risk factors may include environmental factors, genetic/chromosomal abnormalities, and parental age.^12,13^ Medical co-morbidities include, but are not limited to, psychiatric disorders, gastrointestinal disorders, and seizures.^14^ Characteristic symptoms may include behavioral difficulties, developmental and cognitive delays, and psychological problems, all ranging in severity from mild to severe.^15^ Countless available treatments aim to target various symptoms^16^ through behavioral and family-centered means, augmentative and alternative communication (AAC), facilitated communication, and many other treatment models.^17^

Early diagnosis of ASD, i.e., by age 2 years, was found to be possible based on parent interviews and guided observation, while clinical judgment was twice as accurate.^18^ Further, researchers found early diagnosis to remain significantly constant over the years, and, therefore, it is considered important for potentially optimizing intervention outcomes.^18^ A recent study by Fusaro and colleagues^19^ indicated that casual home videos, such as those uploaded by parents to YouTube, could be used for early recognition of ASD. Of note, non-clinicians were able to classify symptoms with a surprising degree of accuracy by analyzing behaviors of young children via the Autism Diagnostic Observation Schedule-Generic (ADOS). Findings suggest that a portion of the diagnostic work can be accomplished successfully by non-clinicians.^19^ Family members have the potential of comparing their infant’s or child’s behaviors to those seen on YouTube and, based on those comparisons, seek professional guidance or feel reassured not to pursue it. However, the variance of behaviors and symptomatology associated with ASD are vast. This, combined with the plethora of non-reviewed videos, could hinder nonprofessionals’ abilities to make predictions by matching the behaviors of one child with another without context.

The aim of our study was to determine the sources and content of popular videos about ASD uploaded to YouTube. Because YouTube is ranked number one for having the highest average US traffic flow with an estimated 1 billion “unique monthly visitors,”^20^ it has the potential of assisting in early identification of ASD and promoting available sources of information (i.e., CDC, NIH) and family and caregiver resources. Further, as a free, public platform (that is not reviewed), it is important to determine the source of the videos and how much of this significant information related to ASD is accurately and completely conveyed.

## Method and Materials

### Procedures

For this cross-sectional study, the 100 most frequently accessed videos on the popular video sharing platform YouTube were categorized by source of upload and examined for content. For the purpose of this study, we determined popularity of videos by number of views, using the filter tool. Videos featured children and adults with ASD, ranging in ages from 1 year; 6 months through over 65 years.

### Measures

The following descriptive information for each of the 100 most viewed videos was recorded: URL, date of upload, source of upload (i.e., personal, professional, television based clip, and internet based clip), length of video, number of “likes” (i.e., thumbs up) and “dislikes” (i.e., thumbs down), and whether the focus of video was on the parents or children.

The following information was recorded relative to accuracy and representativeness of facts: the approximate age of child featured and whether or not the child was alone in the video.

Aspects of social communication, behaviors, interactions, and co-morbidities featured in each video were documented as a Yes or NO to the following questions: Is the child *interacting* with anyone (i.e., looking at, exchanging toys with, talking to anyone)?; Is the child playing with toys appropriately?; Is the child engaged in repetitive (stereotypic) behaviors (e.g., spinning wheels, turning over blocks, repetitive actions on objects that are not “play behaviors”)?; Is the child screaming, yelling, or the like?; Is the child injuring him/herself? Is there mention of sensory problems?

Next, information on risk factors was garnered through mention of any of the following: other siblings and/or family members with ASD; family members with communication disorders (CD) (i.e., other than ASD: speech delay, language delay, low IQ, etc); parents’ ages; difficulty with conception and/or diagnosed infertility; vaccination history.

Finally, information on treatment services for the child and family were noted in terms of whether or not therapy was being depicted in the video, and if there was mention of the following types of therapy/intervention: speech-language therapy; applied behavior analysis (ABA) or other behavior therapy; psychological therapy; other types of therapy (animal, art, herbs, sounds/Tomatis, etc), if a special diet was put into practice (e.g., gluten free, etc), and if there was mention of the child attending a specialized school.

Additionally noted were the following para-therapeutic aspects of ASD: ease of services provided to the family; use of support groups; the economic and/or emotional toll that the disorder has taken on the family; additional sources of information for the viewer; and resources for additional services and support for the viewer of the video.

### Data analyses

Descriptive and inferential statistics were used to analyze the videos and their related features. Descriptive statistics included calculations of frequency, mean, ranges, and percentages. Confidence intervals of proportions were calculated using UCSF Clinical and Translational Science Institute’s calculator (available at http://www.sample-size.net/confidence-interval-proportion/). To determine if a relationship exists between the length of a video and its popularity (i.e., number of views), Pearson correlation coefficient was calculated in Microsoft Excel^®^ 2008 for Mac version 12.3.6 (2007) and *P* values were calculated via Social Science Statistics (Stangroom 2017 available at http://www.socscistatistics.com/pvalues/pearsondistribution.aspx) at 0.01 level of significance.

## Results

A total of 100 of the most viewed videos available on YouTube were analyzed. Forty-three videos were classified as personal, 1 was professional, created by a clinical psychologist, 39 were excerpts of television shows, and 17 were internet-based videos. Videos ranged in length from 0:30–24:29 (mean length 7;39). Number of views totaled 121 355 271, ranging from 19 921–6 676 409 (mean 1 213 553) (Supplementary file 1). Number of views was not correlated with the length of the video (Pearson’s *r* = 0.095, *P* = 0.35). (See Table 1 for video lengths, number of views for each of the four types of videos, and correlations). (Note, numbers of “likes” i.e., thumbs up and “dislikes” i.e., thumbs down were not analyzed due to incomplete data i.e., not all viewers rated the video). All 100 videos were coded, and 10% of the videos were coded by a second rater. Cohen’s unweighted kappa scores were calculated for each coded variable to determine inter-rater reliability. Scores ranged from 0.61–1.0, with an average of 0.98 (SD 0.08), indicating overall strong interrater reliability. See Table 2 for number of videos that depicted each coded variable.

## Discussion

We sought to determine the source of upload and qualitative aspects of information on ASD available to viewers of videos on YouTube, as it is the most popular video upload internet platform. Access to publicly uploaded information has the inherent risk of questionable quality and misinformation. The proliferation of various mainstream media sources (e.g., publications, news shows, video uploads, feature films, etc.) may present unsubstantiated facts, limited information, or biased opinions, which may influence the creators of these YouTube videos and prevent early detection (and hence, early intervention) for interested viewers. In health matters, such as ASD, social media has the potential to provide an early alarm for viewers, or reassurance, both of which should still lead the viewer to a professional. Interestingly, the most frequently viewed videos on ASD were personal videos and television show clips, which, combined, accounted for over 85% of the number of views. Internet based videos accounted for less than 15%. Only one video had been uploaded by a professional, and the number of its views accounted for a mere 0.04% of the total. Although multiple videos may aid in portraying the range of presentations of ASD, an increased professional presence is imperative to help parents (and other professionals) understand that there is no one face of autism, but rather it is a multi-faceted disorder that spans a wide spectrum of abilities and challenges. Additionally, research points to many risk factors, as well as many possible interventions. The popularity of video streaming platforms, such as YouTube, provides an easily accessible venue for professionals to upload their videos about the nature of ASD, possible risk factors, and intervention options and for consumers to access them effortlessly and in one place.

### Social communication, behaviors, interactions, and co-morbidities

Overall, the number of videos that represented a person with ASD interacting with someone (or something) was much higher at 75% compared to the number of videos that portrayed a person with ASD alone (5%). Additionally, the number of videos that portrayed a child with ASD engaging in repetitive behaviors (which included inappropriate play) was greater at 73% than those depicting a child playing with toys (appropriately) at 32%. A high number of videos (63%) depicted a person with ASD engaging in screaming or yelling and 32% of the videos showed a person with ASD injuring him/herself. Mention of co-morbid sensory problems accounted for 64% of all videos. These overall figures are somewhat consistent across each of three types of videos (personal, television clips, internet based video) and not applicable for the one professional video (see Table 2). The accurate depiction in the videos of symptoms and behaviors that may lead the viewer to seek professional guidance would be useful, given that early identification of infants with ASD is crucial for the early provision of treatment. This is important, given that early intervention has the potential of optimizing therapeutic outcomes.^21^ Additionally, as reported by the National Outcomes Measurement System (NOMS) of the American Speech Language Hearing Association (ASHA), the greater number of speech-language pathology sessions for a pre-kindergarten child with ASD results in higher gains in functional communication.^22^ These videos have the potential to lead families to seek appropriate intervention earlier.

### Risk factors

Research continues to explore the genetic correlates of ASD, with the hopes that identification of ASD can occur earlier and inroads to effective treatments can be made.^23,24^ Taking into consideration that only one video was uploaded by a professional, the fact that few risk factors were discussed is not surprising. For example, advanced parental age was noted in only 1 of the 100 videos, and no reference was made to fertility problems of parents of children with ASD, two risk factors of ASD that are associated with genetic mutations.^25,26^ Further, although ASD is known to be highly heritable,^27^ only about 10% of the videos mention other family members with ASD or with CD. Not only do these videos provide minimal available data and research into known ASD risk factors, but also of note is that 16% of the videos continue to espouse debunked vaccine-associated risks.

### Information on treatment services

The available treatment services for persons with ASD depend primarily on the severity of the disorder, and can range from use of AAC to traditional communication therapies.^28^ Although 58% of the videos mention speech-language therapy as an intervention, only 23% of the videos depict a speech-language therapy session. ABA was mentioned in 75% of the videos, although the effects of ABA on specific primary characteristics of ASD, such as impaired social communication and language-based skills, are unclear.^29-31^ As increasingly more research is examining neurological aspects that may regulate the behaviors of persons with ASD,^32^ it is important to note that atypical social cognition may be correlated with atypical activation of complex neural structures, despite intervention. Other treatments, such as psychological interventions and mention of alternative approaches (e.g., animal therapy, art therapy, and herbs) accounted for 72% and 75% of the videos, respectively. The therapeutic landscape has become quite varied, especially in the last decade. A thorough treatise on naturalistic developmental behavioral interventions (NDBIs) has been presented, covering their historical evolution as they emerged from incorporating the principles from different theoretical camps to their currently validated parameters.^33^ Mention of sensory diets (e.g., gluten free, casein-free diets) accounted for 15% of the videos. Although many parents report positive outcomes of such diets, clinically controlled studies are needed to determine their efficacy.^34^ It is unclear how the content of so many (i.e., ~75%) of the most popular videos influences the decisions of parents of children of ASD when choosing a treatment approach. It has been reported that there is little communication between parents and pediatricians about possible intervention options for children with ASD.^35^ Further, pediatricians and parents often disagreed about alternative treatment options, causing parents to seek information elsewhere. A platform such as YouTube could be used by professionals to discuss the “safety and efficacy”^35^ of such treatments, a perspective missing from parent-professional exchanges (and from the most viewed videos on YouTube).

Schooling models range from children with ASD learning in inclusive general education classrooms to attending schools specialized in educating children with ASD. Thirty-two percent of the videos mention attendance at a special school (i.e., not a general education setting). Helpful information to include within these videos may be specific needs or symptoms that may qualify a child with ASD to enroll in a non-mainstream setting. Children should be provided with optimal educational resources to meet individual needs.

### Para-therapeutic aspects of ASD

Support groups for parents of children with ASD have been noted to facilitate parent-child interactions and increase parents’ skills and knowledge.^36^ Only 43% of the videos mention support groups, yet 54% mention the economic toll the disorder has inflicted on the family resources, and 61% mention the emotional toll the diagnosis has taken on the family. Of the videos, 30% mention or direct the viewer to other sources of information on ASD, and only 22% mention resources for additional services and supports. These resources have the potential to greatly influence outcomes of people with ASD and information would be valuable if provided in some way across a higher number of videos.

### Portrayal of ASD

ASD was portrayed negatively in 10% of the videos (7.98% of the personal videos, 15.79% of the television clips, 5.88% of the internet based videos, and the professional video was not deemed to portray ASD negatively). These videos have the potential to both bring public awareness as well as skew perceptions of ASD. Although appropriate and positive information about ASD was conveyed in the majority of videos, all videos, including those that highlight negative attributes of ASD, would benefit the viewers more by emphasizing a more balanced view of the disorder.

### Limitations

This study is limited in that the design was cross sectional, which limits the ability to generalize findings. This is especially true given the transitional nature of YouTube. In addition, this study only focused on one popular video-sharing site versus including other popular social media platforms. Lastly, the videos included in this sample were only those conducted in English.

In summary, our study aimed to determine the source of upload and content of the most popular 100 videos on YouTube about ASD. An open unregulated information-sharing platform like YouTube has an enormous potential for disseminating highly valuable, accurate, evidence-based information to millions of viewers. However, our findings show that 99% of the most popular videos are created and uploaded by non-professionals, and the content of these videos is limited. Professionals, such as physicians, speech-language pathologists, educators, psychologists, occupational therapists, and physical therapists, have an opportunity to provide easily accessible, online education and an obligation to provide high quality, valid, up-to-date information to viewers via this preferred platform to ensure early detection of signs, symptoms, and resources. Viewers must also be made aware that one video is unlikely to provide all of the information needed. Sources for additional information and available resources (e.g., links to credible sources of information such as the CDC and NIH) should be included in all professionally created and uploaded videos.

## Ethical approval

The Institutional Review Boards of William Paterson University of New Jersey and Marymount Manhattan College do not review studies that do not involve human subjects.

## Competing interests

The authors declare that they have no conflicts of interest.

## Authors’ contributions

BK and CB conceptualized the study and designed data collection methodology. BK, CB, and MKS led manuscript development. MKS conducted data analysis. AC was responsible for data collection and assisted with manuscript development.

## Supplementary Materials

Supplementary file 1 contains Tables S1-S4.

## Acknowledgments

No financial support was received for this project.

**Table 1:** Video Analyses^a^

**Source of upload**	**Total number of views**	**Mean number of views (range)**	**Mean length of video in seconds (minutes; seconds)**	**Range of length of videos**	**Pearson’s r**
All videos, n = 100	121 355 271	1 213 553 (19 921 – 6 676 409)	459 (7;39)	0:30–24:29	0.095 (*P* = 0.35)
Personal videos (parent video), n = 43 (34)	47 580 901 (39.2%)	1 106 533 (19 921-4 352 050)	363 (6;03)	1;09–19;57	-0.285 (*P* = 0.06)
Professional, n =1	48 397 (0.04%)	48 397	226 (3;16)	3;16	NA
Television show clip, n =39	55 636 158 (45.85%)	1 426 568 (103 292-6 676 409)	431 (7;11)	0:30–21;32	-0.173 (*P* = 0.30)
Internet based video, n =17	18 089 814 (14.91%)	1 064 107 (342 017-4 137 053)	778 (12;58)	2;00–24;29	-0.038 (*P* = 0.13)

^a^Correlations of number of views and video lengths for each of the four types of videos categorized by source of upload.

**Table 2:** Content analysis of videos on ASD

**Content parameter**	Total No. of videos n = 100 n (%, 95% CI)	Personal Videos n = 43 n (%, 95% CI)	Professional n =1 n*	Television show clip n =39 n (%, 95% CI)	Internet based video n =17 n (%, 95% CI)
Public Service Announcement	34 (34, 25-44)	10 (23.26, 11.8-38.6)	1	13 (33.33, 19.1-50.2)	10 (58.82, 32.9-81.6)
Child is alone in the video	5 (5, 02-11)	4 (9.30, 2.6-22.1)	0	1 (2.56, 0.1-13.5)	0 (0, 0-19.5**)
Child is interacting with someone/thing	75 (75, 65-83)	31 (72.09, 56.3-84.7)	0	33 (84.62, 69.5-94.1)	11 (64.71, 38.3-85.8)
Child is playing with toys	32 (32, 23-42)	9 (20.93, 10.0-36.0)	0	17 (43.59, 27.8-60.4)	6 (35.29, 14.2-61.7)
Child is engaged in repetitive behaviors	73 (73, 63-81)	33 (76.74, 61.4-88.2)	0	32 (82.05, 66.5-92.5)	8 (47.06, 23.0-72.2)
Child is screaming/yelling	63 (63, 53-73)	30 (69.77, 53.9-82.8)	1	23 (58.97, 42.1-74.4)	9 (52.94, 27.8-77.0)
Child is injuring him/herself	32 (32, 23-42)	16 (37.21, 23.0-53.3)	0	13 (33.33, 19.1-50.2)	3 (17.65, 03.8-43.4)
Mention of sensory problems	64 (64, 54-73)	28 (65.12, 49.1-79.0)	0	24 (61.54, 44.6-76.6)	12 (70.59, 44.0-89.7)
Mention of siblings with ASD	9 (9, 04-16)	2 (4.65, 0.6-15.8)	0	5 (12.82, 04.3-27.4)	2 (11.76, 01.5-36.4)
Mention of family members with CD	10 (10, 05-18)	3 (6.98, 1.5-19.1)	0	5 (12.82, 04.3-27.4)	2 (11.76, 01.5-36.4)
Mention of infertility	1 (1, 0-5.4)	0 (0, 0-8.2**)	0	0 (0, 0-9.0**)	1 (5.88, 0.1-28.7)
Mention of parent age	0 (0-3.6**)	0 (0, 0-8.2**)	0	0 (0, 0-9.0**)	0 (0, 0-19.5**)
Mention of vaccines	16 (16, 09-25)	6 (13.95, 5.3-27.9)	0	6 (15.38, 05.9-30.5)	4 (23.53, 06.8-49.9)
Mention of SLP/SLT	58 (58, 48-68)	22 (51.16, 35.5-66.7)	1	25 (64.10, 47.2-78.8)	10 (58.82, 32.9-81.6)
Therapy session (speech-language therapy)	23 (23, 15-33)	6 (13.95, 5.3-27.9))	1	11 (28.21, 15.0-44.9)	5 (29.41, 10.3-56.0)
Mention of treatment (ABA or other)	75 (75, 65-83)	31 (72.09, 56.3-84.7)	1	30 (76.92, 60.7-88.9)	13 (76.47, 50.1-93.2)
Mention of treatment (psychological)	72 (72, 62-81)	28 (65.12, 49.1-79.0)	1	30 (76.92, 60.7-88.9)	13 (76.47, 50.1-93.2)
Mention of treatment (other: animal, art, herbs)	75 (75, 65-83)	30 (69.77, 53.9-82.8)	1	31 (79.49, 63.5-90.7)	13 (76.47, 50.1-93.2)
Mention of special diets	15 (15, 09-24)	7 (16.28, 6.8-30.7)	0	5 (12.82, 04.3-27.4)	3 (17.65, 03.8-43.4)
Mention of child attending a special school	32 (32, 23-42)	15 (34.88, 21.0-50.9)	1	15 (38.46, 23.4-55.4)	1 (5.88, 0.1-28.7)
Mention of ease of services provided to the family	38 (38, 29-48)	15 (34.88, 21.0-50.9)	1	15 (38.46, 23.4-55.4)	7 (41.18, 18.4-67.1)
Mention of support groups	43 (43, 33-53)	17 (39.53, 25.0-55.6)	1	17 (43.59, 27.8-60.4)	8 (47.06, 23.0-72.2)
Mention of economic toll	54 (54, 44-64)	21 (48.84, 33.3-64.5)	1	22 (56.41, 39.6-72.2)	10 (58.82, 32.9-81.6)
Mention of emotional toll	61 (61, 51-71)	23 (53.49, 37.7-68.8)	1	26 (66.67, 49.8-80.9)	11 (64.71, 38.3-85.8)
Mention of other sources of information for the viewer	30 (30, 21-40)	12 (27.91, 15.3-43.7)	1	11 (28.21, 15.0-44.9)	6 (35.29, 14.2-61.7)
Mention of resources for additional services, supports, etc	22 (22, 14-31)	10 (23.26, 11.8-38.6)	1	7 (17.95, 07.5-33.5)	4 (23.53, 06.8-49.9)
ASD is portrayed negatively	10 (10, 05-18)	3 (6.98, 1.5-19.1)	0	6 (15.38, 05.9-30.5)	1 (5.88, 0.1-28.7)

*Percentages not calculated. ** 97.5% CI, one-sided.
