# Supplementary material for: Sources and content of popular online videos about autism spectrum disorders
Source: Health Promot Perspect. 2017 Sep 26;7(4):238–44. doi: 10.15171/hpp.2017.41 (PMC5647360; doi:10.15171/hpp.2017.41)
Supplement: Supplementary file 1 [file hpp-7-238-s01.pdf]

Health Promotion Perspectives, 2017, 7(4), 238-244
doi: 10.15171/hpp.2017.41

Table S1. Raw data of personal videos *n* = 43

URL of video	Date of upload	# of views	Length of video	seconds	# of thumbs up	# of thumbs down
https://www.youtube.com/watch?v=IIX4a_6P8J0	07/06/2016	19,921	4:21	261	60	8
https://www.youtube.com/watch?v=-Oyj89ynMNA	07/08/2016	148,084	19:54	1,194	1,445	121
https://www.youtube.com/watch?v=iipltmM5BFs	08/22/2013	179,361	19:57	1,197	367	66
https://www.youtube.com/watch?v=jXvozvJSCNU	01/03/2012	302,150	8:26	506	284	56
https://www.youtube.com/watch?v=maLzqjYx1BM	04/02/2016	318,050	3:16	196	3,911	156
https://www.youtube.com/watch?v=PPWL5yimhyg	08/20/2009	372,280	3:02	182	568	176
https://www.youtube.com/watch?v=Iu7C5clA4q0	09/19/2009	372,584	6:26	386	725	89
https://www.youtube.com/watch?v=LdsDRIRXYww	02/08/2015	379,868	8:44	688	1,627	168
https://www.youtube.com/watch?v=uAyhz2m-UuQ	11/09/2006	404,896	5:17	317	0	0
https://www.youtube.com/watch?v=nQxDG4LD1Eo	11/17/2007	413,626	1:09	69	108	36
https://www.youtube.com/watch?v=8jrqpn60d4A	09/29/2010	418,642	7:05	425	1,091	159
https://www.youtube.com/watch?v=CV2Zm2EZKuw	04/21/2007	451,476	9:34	574	404	54
https://www.youtube.com/watch?v=kdKVUfsfyG0	06/30/2014	461,791	3:12	192	538	227
https://www.youtube.com/watch?v=plPNhooUUuc	08/18/2010	508,275	3:16	196	0	0
https://www.youtube.com/watch?v=RGSYBdvUF9w	05/01/2013	539,555	2:13	133	15,944	265
https://www.youtube.com/watch?v=SjQcxATh1vI	10/19/2008	556,849	3:51	231	1,208	178
https://www.youtube.com/watch?v=T3PwG36iKH8	03/31/2009	597,477	2:35	155	2,140	142
https://www.youtube.com/watch?v=vKWuzklXUk0	02/25/2013	636,042	4:06	246	5,951	340
https://www.youtube.com/watch?v=MDwXqGjohGg	07/28/2013	659,745	5:16	316	6,389	168
https://www.youtube.com/watch?v=ze_Hlkz8dDs&t=9s	02/19/2009	693,440	8:16	496	3,411	1,186
https://www.youtube.com/watch?v=OgfZ8jFxvvw	01/08/2013	764,203	4:56	296	1,359	229
https://www.youtube.com/watch?v=ACLV9hytZb0	01/04/2014	784,120	2:33	153	772	57
https://www.youtube.com/watch?v=Kc12f6MtQmI	01/10/2012	799,759	11:36	696	1,931	1,003
https://www.youtube.com/watch?v=mc1H0aVqn20	04/02/2008	1,029,526	5:56	356	0	0
https://www.youtube.com/watch?v=spuMFceTZGo&t=2s	09/20/2014	1,059,694	2:15	135	2,549	456
https://www.youtube.com/watch?v=G7kHSOgauhg&t=4s	08/20/2006	1,066,382	3:11	191	2,071	149
https://www.youtube.com/watch?v=j4PTf7LgsIE&t=1s	03/14/2012	1,107,733	2:48	168	4,008	713

Health Promotion Perspectives, 2017, 7(4), 238-244
doi: 10.15171/hpp.2017.41

https://www.youtube.com/watch?v=JsJ154jWRsU&t=4s	08/20/2009	1,152,659	7:07	427	3,530	436
https://www.youtube.com/watch?v=ukDKrwoL36g	01/22/2016	1,264,924	2:07	127	9,115	148
https://www.youtube.com/watch?v=iROplC_gois	02/01/2015	1,346,675	10:47	647	8,896	476
https://www.youtube.com/watch?v=ZCU8wxCdKIE	01/16/2016	1,349,108	19:02	1,142	26,703	579
https://www.youtube.com/watch?v=QHC0FzywHGY&t=12s	01/17/2013	1,378,525	6:10	370	13,319	240
https://www.youtube.com/watch?v=rlNCz-SF-5I	03/17/2015	1,398,982	5:54	354	2,607	1,261
https://www.youtube.com/watch?v=JnylM1hI2jc	01/14/2007	1,416,176	8:36	516	6,532	432
https://www.youtube.com/watch?v=rbgUjmeC-4o	03/29/2007	1,476,809	8:05	485	11,635	1,236
https://www.youtube.com/watch?v=IcS2VUoe12M	02/22/2012	1,581,426	2:39	159	0	0
https://www.youtube.com/watch?v=OvUFh_0xoIs	02/21/2010	1,939,030	6:43	403	4,362	384
https://www.youtube.com/watch?v=uud3CtpMbEA	08/16/2011	1,965,934	5:24	324	12,908	2,548
https://www.youtube.com/watch?v=2UWV1dlueKc	11/20/2013	2,173,942	3:38	218	78	17
https://www.youtube.com/watch?v=KmDGvquzn2k	05/24/2012	2,732,418	2:18	138	13,503	318
https://www.youtube.com/watch?v=AR5Y9R6kBXw	05/09/2016	3,257,233	1:14	74	12,290	377
https://www.youtube.com/watch?v=QeKHRJ2Pm0Y	02/12/2014	3,749,481	1:39	99	8,973	324
https://www.youtube.com/watch?v=EKomAGJ24fQ	12/07/2010	4,352,050	2:51	171	2,555	1,271

Table S2. Raw data of professional videos *n* = 1

URL of video	Date of upload	# of views	Length of video	seconds	# of thumbs up	# of thumbs down
https://www.youtube.com/watch?v=1j8wK_IkMcM	12/02/2012	48,397	3:46	226	25	4

Table S3. Raw data of television based clips *n* = 39

**URL of video**	**Date of upload**	**# of views**	**Length of video**	**seconds**	**# of thumbs up**	**# of thumbs down**
https://www.youtube.com/watch?v=S0w6carvS8k&t=17s	03/24/2015	103,292	20:49	1,249	1,107	26

Health Promotion Perspectives, 2017, 7(4), 238-244
doi: 10.15171/hpp.2017.41

https://www.youtube.com/watch?v=o3P6wVUH0pc	01/19/2015	337,524	8:19	659	5,229	809
https://www.youtube.com/watch?v=V-c50HNnPg0	07/24/2011	345,958	6:41	401	860	44
https://www.youtube.com/watch?v=BEqrIbzskp4	11/07/2014	349,071	2:22	142	680	89
https://www.youtube.com/watch?v=3IOthQJwKzQ	11/25/2015	385,016	4:49	289	6,562	452
https://www.youtube.com/watch?v=zrIM2hwrLoc	10/19/2007	396,525	7:28	448	1,954	259
https://www.youtube.com/watch?v=34xoYwLNpvw	08/12/2009	397,774	9:51	591	1,738	25
https://www.youtube.com/watch?v=wKlMcLTqRLs	04/28/2014	408,493	15:34	934	3,575	449
https://www.youtube.com/watch?v=6oEtF8FdqpA	05/07/2009	409,088	3:59	239	2,162	375
https://www.youtube.com/watch?v=dgwYukPReKw	04/17/2012	419,192	9:00	540	1,582	62
https://www.youtube.com/watch?v=vqbXPfaN_VM	04/11/2007	430,862	5:23	323	565	20
https://www.youtube.com/watch?v=FuWWie1DlJY	11/30/2007	475,895	3:56	236	268	36
https://www.youtube.com/watch?v=zVgPlURSad8	03/21/2011	522,367	13:08	788	744	78
https://www.youtube.com/watch?v=qGW4a96GqGc	10/23/2008	573,497	3:10	190	2,560	1,483
https://www.youtube.com/watch?v=I7lAN1zno0w	04/05/2013	580,349	4:18	258	1,932	43
https://www.youtube.com/watch?v=2wt1IY3ffoU	02/07/2008	595,209	21:32	1,292	2,815	81
https://www.youtube.com/watch?v=dWdy6Iy0FK8&t=7s	08/12/2014	610,932	4:28	268	2,923	30
https://www.youtube.com/watch?v=6iYT8p2i_nU&t=3s	11/21/2013	741,861	4:11	251	6,189	436
https://www.youtube.com/watch?v=8Vs6R5YZQ3c	09/22/2006	746,851	8:10	490	1,533	49
https://www.youtube.com/watch?v=p6cOp6EDFlI	03/06/2006	767,534	2:47	167	1,812	51
https://www.youtube.com/watch?v=LGj47A14naM	02/20/2015	775,678	18:27	1,107	3,098	93
https://www.youtube.com/watch?v=BoqnPZXLGVA	04/27/2016	922,072	13:20	800	13,933	545
https://www.youtube.com/watch?v=T8V_0LUCgmk	11/10/2016	1,005,234	10:21	621	3,854	44
https://www.youtube.com/watch?v=UTx12y42Xv4	04/02/2016	1,025,615	3:33	213	27,994	1,246
https://www.youtube.com/watch?v=VULKzVZCso0	01/29/2015	1,035,072	4:37	277	5,485	64
https://www.youtube.com/watch?v=u2obRVpEl4Q	12/03/2012	1,052,432	1:46	106	1,272	64
https://www.youtube.com/watch?v=TqEZQxP1azM	05/10/2015	1,214,113	2:04	124	5,863	857
https://www.youtube.com/watch?v=1MwuJy07lQY	01/04/2011	1,233,301	12:02	722	1,824	5,008
https://www.youtube.com/watch?v=cXClmauUTTs	10/20/2016	1,280,033	1:06	66	16,304	329
https://www.youtube.com/watch?v=mTGgwF5ms-g&t=1s	03/24/2016	1,383,338	7:06	426	10,168	340
https://www.youtube.com/watch?v=ycCN3qTYVyo&t=5s	04/09/2014	2,260,978	1:08	68	2,626	61

Health Promotion Perspectives, 2017, 7(4), 238-244
doi: 10.15171/hpp.2017.41

https://www.youtube.com/watch?v=ycCN3qTYVyo&t=7s	04/09/2014	2,262,161	1:08	68	2,628	62
https://www.youtube.com/watch?v=4g5jtGqHJEw	02/08/2008	2,278,624	0:30	30	0	0
https://www.youtube.com/watch?v=OR36jrx_L44	01/15/2012	3,065,043	13:49	829	19,223	877
https://www.youtube.com/watch?v=1fw1CcxCUgg	02/27/2006	3,618,059	2:10	130	7,511	203
https://www.youtube.com/watch?v=a34qMg0aF6w	04/29/2016	3,716,926	7:00	420	47,057	257
https://www.youtube.com/watch?v=Lr4_dOorquQ	03/31/2016	4,979,631	1:24	84	0	0
https://www.youtube.com/watch?v=vNZVV4Ciccg	05/25/2011	6,254,149	9:52	592	38,076	698
https://www.youtube.com/watch?v=-F_W_zl61bI	04/10/2014	6,676,409	6:21	381	62,749	990

Table S4. Raw data of internet-based videos *n* = 17

URL of video	Date of upload	# of views	Length of video	seconds	# of thumbs up	# of thumbs down
https://www.youtube.com/watch?v=G_BS_LkhRmk	02/11/2011	342,017	5:59	359	1,994	559
https://www.youtube.com/watch?v=jQ95xlZeHo8	11/21/2014	379,867	6:12	372	6,699	315
https://www.youtube.com/watch?v=TL5GHMEjzt8	07/08/2008	392,109	6:17	377	4,178	118
https://www.youtube.com/watch?v=AbeyIG7Fz8s	02/21/2010	396,830	3:53	233	1,979	95
https://www.youtube.com/watch?v=idOBGNTz-6E	07/14/2008	403,313	2:26	146	0	0
https://www.youtube.com/watch?v=89KnHRLz7EQ	07/12/2010	444,333	4:46	286	356	66
https://www.youtube.com/watch?v=REL4Sc2iw1Y	08/31/2016	476,315	3:57	237	14,942	124
https://www.youtube.com/watch?v=o65l1YAVaYc	01/19/2014	505,800	7:56	476	8,586	6,207
https://www.youtube.com/watch?v=7pN6ydLE4EQ	02/07/2012	507,689	10:36	6,336	1,350	147
https://www.youtube.com/watch?v=MCEqfvSr0Fc&t=5s	03/11/2013	667,478	3:24	204	1,683	193
https://www.youtube.com/watch?v=FO1W7iXTSQE	05/21/2012	727,308	13:50	830	1,531	902
https://www.youtube.com/watch?v=cBOSr7JK8OA	04/01/2014	791,226	3:55	235	4,918	121
https://www.youtube.com/watch?v=SJ93HTeKc98	06/06/2012	816,203	8:36	516	2,255	204
https://www.youtube.com/watch?v=lbXjW-cX9kQ&t=7s	06/17/2011	1,211,513	14:07	847	4,131	351
https://www.youtube.com/watch?v=r8k5IsGV7ZM&t=3s	04/01/2008	2,146,306	3:08	188	495	179
https://www.youtube.com/watch?v=8af0QPhJ22s	07/16/2014	3,744,454	24:29:00	1,469	47,878	2,131
https://www.youtube.com/watch?v=oMN2PeFama0	04/02/2016	4,137,053	2:00	120	71,062	5,035
